# Epigenetics of alcohol use disorder—A review of recent advances in DNA methylation profiling

**DOI:** 10.1111/adb.13006

**Published:** 2021-02-03

**Authors:** Martha J. Longley, Jisoo Lee, Jeesun Jung, Falk W. Lohoff

**Affiliations:** ^1^ Section on Clinical Genomics and Experimental Therapeutics National Institute on Alcohol Abuse and Alcoholism, National Institutes of Health Bethesda Maryland USA

**Keywords:** addiction, alcohol abuse, alcohol dependence, alcohol use disorder, DNA methylation, epigenome‐wide association study

## Abstract

Alcohol use disorder (AUD) is a major contributor to morbidity and mortality worldwide. Although there is a heritable component, the etiology of AUD is complex and can involve environmental exposures like trauma and can be associated with many different patterns of alcohol consumption. Epigenetic modifications, which can mediate the influence of genetic variants and environmental variables on gene expression, have emerged as an important area of AUD research. Over the past decade, the number of studies investigating AUD and DNA methylation, a form of epigenetic modification, has grown rapidly. Yet we are still far from understanding how DNA methylation contributes to or reflects aspects of AUD. In this paper, we reviewed studies of DNA methylation and AUD and discussed how the field has evolved. We found that global DNA and candidate DNA methylation studies did not produce replicable results. To assess whether findings of epigenome‐wide association studies (EWAS) were replicated, we aggregated significant findings across studies and identified 184 genes and 15 gene ontological pathways that were differentially methylated in at least two studies and four genes and three gene ontological pathways that were differentially methylated in three studies. These genes and pathways repeatedly found enrichment of immune processes, which is in line with recent developments suggesting that the immune system may be altered in AUD. Finally, we assess the current limitations of studies of DNA methylation and AUD and make recommendations on how to design future studies to resolve outstanding questions.

## Graphical abstract

The number of studies investigating DNA methylation patterns in individuals with alcohol use disorder (AUD) has grown steadily over the past decade. Here, we review research on AUD and DNA methylation and identify CpG sites that have been most frequently replicated.

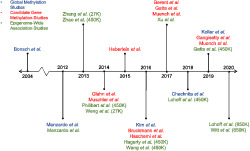

## 1 INTRODUCTION

Alcohol use disorder (AUD) is a disease characterized by an inability to stop or control alcohol use despite negative social, occupational, or health consequences. AUD affects over 100 million individuals worldwide and is associated with significant morbidity and mortality.^1^ Nearly 100 million disability‐adjusted life years, which are estimated years lost due to illness, disability, or early death, were attributed to alcohol misuse in 2016.^1^ Despite the widespread prevalence of the disease, there are few effective interventions for prevention and treatment, and little is known about its underlying etiology. This may be due in part to the wide range of alcohol‐related phenotypes that AUD can represent. Most studies in this review classified AUD according to the Diagnostic and Statistical Manuel of Mental Disorders, Fourth Edition (DSM‐IV), which divides AUD into *alcohol dependence* based upon satisfaction of three or more criteria relating to physical or psychological dependence and *alcohol abuse*, which requires satisfaction of at least one criterion involving alcohol use despite physical or mental damage to the individual.^2^ The most recent edition of the manual, the DSM‐5, groups the same criteria (with the exception of one criterion) into AUD, which is classified as mild, moderate, or severe. Both across and within these diagnostic categories, AUD can represent many combinations of symptoms, symptom severity, and alcohol consumption patterns, leading to a wide range of alcohol‐related phenotypes.

Despite the phenotypic variability in AUD, studies have consistently found genetic factors to influence an individual's risk of developing the disorder. For example, family and twin studies have shown AUD to be 50%–70% heritable.^3^, ^4^, ^5^, ^6^ Although genome‐wide association studies to date have succeeded in identifying some variants associated with AUD, they are not able to fully explain the complex etiology of AUD.^7^, ^8^ Epigenetic modifications, which are changes in gene expression that are not attributable to changes in DNA sequence, can contribute to gene expression and thus may provide insight into physiological causes and consequences of AUD. Epigenetic modifications can also be influenced by genetic variants and environmental exposures and have been shown to play a role in determining when and to what extent gene transcription occurs.^9^

**FIGURE 1:**
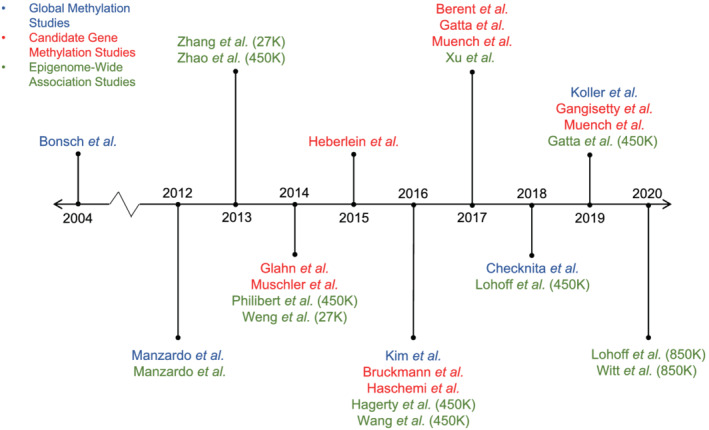
Timeline of studies on AUD and DNA methylation

A growing recognition of the importance of epigenetics and advances in assays for DNA methylation, a well‐known form of epigenetic modification, have led to a rapid rise in the number of studies investigating AUD and DNA methylation in the past decade (Figure 1). In this review, we focus on DNA methylation, as it is the best studied form of epigenetic regulation in human studies of AUD. DNA methylation refers to methylation at the fifth carbon in cytosine bases and most often occurs in CpG dinucleotides, which consist of a cytosine followed by a guanine. DNA methylation is typically carried out by proteins in the DNA methyltransferase family.^10^ On the other hand, demethylation can occur either through deamination or removal of methylated bases via activation‐induced deaminases or ten–eleven translocations, respectively. The removed or deaminated bases are then replaced through the base excision repair pathway.^11^ Though the biological roles of DNA methylation are not fully understood, it has several frequently discussed roles in regulating gene expression. DNA methylation in the promoter region has been shown to promote or inhibit transcription factor binding, thereby activating or silencing transcription, respectively.^10^ DNA methylation in the gene body has been positively correlated with gene expression and intergenic DNA methylation may also affect transcription.^10^, ^12^ DNA methylation can also recruit methyl CpG binding proteins that in turn recruit histone deacetylase complexes that repress gene expression.^11^ Demethylation, or the replacement of a 5‐methylcytosine with a cytosine, likely has the opposite effect as DNA methylation and has been associated with some diseases.

Many standard DNA methylation assays do not distinguish between methylation and hydroxymethylation, which means that values that are being recorded as differential methylation may be due to varying degrees of differential hydroxymethylation rather than methylation. Structurally, the two are similar—hydroxymethylation refers to the covalent addition of a hydroxymethyl group, rather than a methyl group, to the C5‐position of cytosine in CpG dinucleotides. Functionally, however, they may be different. Hydroxymethylation is not fully understood, but it is common in the brain and may play a role in DNA repair or transcription factor binding or may be a transition state of DNA methylation.^12^, ^13^, ^14^ Our review identified only one epigenome‐wide association study (EWAS) that used array technology and also differentiated between methylation and hydroxymethylation. In this study, some of the top hits had higher proportions of hydroxymethylated sites than methylated CpG sites, which highlights the importance of distinguishing between the two types of methylation as the signal may at times be a hydroxymethylation rather than methylation signal.

Specific to AUD, alcohol has been shown to alter DNA methylation patterns in rodents, coinciding with increases in voluntary alcohol administration that mimic a transition from light “social” drinking to excessive alcohol consumption in humans.^15^, ^16^, ^17^, ^18^, ^19^ Thus, DNA methylation could play a role in the biological consequences of alcohol consumption and may also contribute to the neurobiological architecture of AUD pathology. In this review, we summarize how our understanding of the associations between DNA methylation and the disease‐specific phenotype AUD has evolved. We performed a search in PubMed using the terms “DNA methylation” and “alcohol abuse,” “alcohol dependence,” or “alcohol use disorder” from January 2000 to April 2020. We reviewed all papers that resulted from this search and included in this review original articles on DNA methylation in human specimens (*n* = 27). We excluded studies for which the DNA methylation and AUD data were not in the same individuals, for example, studies of maternal AUD and offspring DNA methylation patterns. Though included in our discussion, we also excluded studies on alcohol consumption from our primary analysis. Based on our findings, we discuss the types of DNA methylation studies, including global DNA methylation, candidate gene methylation, and EWAS, and what we have learned from each form of inquiry. We also aggregate available EWAS data to identify molecular targets and pathways that had significant differential methylation in two or more studies. We used this as a measure of replication in light of generally small sample sizes in currently available EWAS data. Finally, we discuss challenges involved in interpreting DNA methylation data in AUD and how these may be resolved in the future.

## 2 ADVANCES IN DNA METHYLATION PROFILING STUDIES AND DISCREPANCIES IN FINDINGS

The number of studies investigating DNA methylation and AUD has grown steadily over the past decade: between 2004 and 2015, only 10 studies had been published on the relationship between DNA methylation and AUD in humans, while 16 have been published in the past 5 years alone (Table 1). Although most studies used DSM‐IV diagnostic criteria, some classified AUD using the Alcohol Use Disorder Identification Test (AUDIT), which is a 10‐item questionnaire that categorizes alcohol use as harmful or hazardous based on alcohol‐related problems and behaviors.^20^ We also included studies that use the Semi‐Structured Assessment for the Genetics of Alcoholism (SSAGA‐II), which is based upon DSM‐III and IV criteria, and the International Classification of Diseases, tenth revision (ICD‐10), which is influenced by but not identical to the DSM‐IV (Table 1 and supporting information Table S1). Five studies in our search examined global DNA methylation or the average methylation of cytosine bases across the genome in cases compared to controls. However, because they provided little insight into the disorder and all studies had small sample sizes, varying assays and differing results, we decided not to include them in the primary analysis.

**TABLE 1:** Epigenome‐wide association studies of alcohol use disorders

Author	Sample	Diagnosis	Tissue	DNA methylation assay	Statistical adjustments	Significant findings
*Studies of peripheral tissue*						
Zhang et al., 2013	63 AD cases and 65 age‐and ethnicity‐matched controls	DSM‐IV	Lymphocytes	HM27K	Did not control for potential confounding variables such as smoking and BMI	1710 CpG sites differentially methylated (1702 hypomethylated and 8 hypermethylated)
Zhao et al., 2013	Alcohol‐dependent patients and their siblings (*n* = 20 Han Chinese individuals)	DSM‐IV	Mononuclear cells	HM450K	Did not control for potential confounding variables such as smoking and BMI	865 hypomethylated and 716 hypermethylated sites identified
Philibert et al., 2014	33 cases and 33 controls (mostly European American males)	SSAGA‐II (treatment for AD)	Mononuclear cells	HM450K	FDR, Bonferroni and batch corrected but not corrected for age, gender, and ethnicity	Significantly different methylation at 56 probes; alcohol‐related methylation reduced during treatment
Weng et al., 2014	10 male Taiwanese cases (healthy at Time Points 1 and 2 and AD at Time Point 3) and 10 male Taiwanese controls (healthy at Time Points 1–3)	DSM‐IV	Blood	HM27K	Included age, alcohol consumption, and betel nut use in the model	149 genes hypermethylated and 51 genes hypomethylated in cases
Xu et al., 2017	256 AA (117 cases with AD‐ND) and 196 EA (103 AD‐ND cases and 93 controls)	DSM‐IV	Blood	Illumina GoldenGate DNA Methylation Array	Adjusted for age, sex, and ancestry proportions, batch effect considered	70 nominally significant CpGs in both EA and AA
Lohoff et al., 2018	Discovery: 23 AUD subjects (16 male and 7 female) and 23 age‐matched healthy controls; NICHD: *n* = 29 cases 29 controls with MDD; 68 AUD and 72 healthy controls	DSM‐IV	Blood and brain	HM450K	Discovery: controlled for age, sex, and neuronal proportion NICHD: controlled for age, sex, and postmortem interval and multiple testing using FDR; age, gender, AIM scores, and scanner types included as nuisance covariates	Five differentially methylated genes in brain after FDR correction
Lohoff et al., 2020	Discovery: 336 AUD and 203 controls First RC: (43 AUD and 43 controls) Second RC: (4301 Scottish) Third RC: (392 Grady Trauma Project) Postmortem Brain 1: 58 individuals with and without MDD Postmortem Brain 2: 23 participants with alcohol dependence/abuse and 23 age‐matched controls	Discovery: First RC: SCID‐IV Second RC: Clinical Questionnaire Third RC: SCID‐IV Postmortem Brain 1: alcohol problem based on clinical interview Postmortem Brain 2: DSM‐IV	Discovery: blood First RC: blood Second RC: blood Third RC: blood Postmortem Brain 1: neuronal and glial tissues Postmortem Brain 2: brain	Discovery: HM850K First RC: Infinium HM850K Second RC: HM850K Third RC: HM850K Postmortem Brain 1: 450K Postmortem Brain 2: 450 K	M‐values corrected for age, sex, relatedness batch and estimated cell counts, and smoking status and pack years of smoking.	4798 cites differentially methylated in AUD
Witt et al., 2020	99 alcohol‐dependent males with severe withdrawal and 95 age‐matched controls	DSM‐IV; CIWA‐Ar > 4	Blood	HM850K	Smoking and age as covariates, Houseman correction and FDR corrected	2876 cytosine‐phosphate‐guanine (CpG) sites between cases and controls as well as 9845 sites that were differentially methylated at time point 1 and 6094 at time point 2. Differential methylation between cases and controls decreased at over 800 CpG sites between Time Points 1 and 2.
*Studies of brain tissue*						
Manzardo et al., 2012	10 AD and 10 gender‐matched controls	DSM‐IV	Postmortem frontal cortex	Roche NimbleGen Human DNA Methylation 2.1M Deluxe Promoter Array	Used Kolmogorov–Smirnov test to predict differential methylation (did not control for potential confounding variables such as BMI, smoking etc.)	No difference in global methylation between AD and controls, AD had higher mean peak scores in 1010 genes, lower mean peak scores in 1270 genes, and exclusive methylation in 618 genes; controls had exclusive methylation in 908 genes.
Hagerty et al., 2016	49 AD and 47 controls; 24 postmortem subjects	DSM‐IV	Postmortem precuneus brain tissue (49 AD and 47 controls) and precuneus and putamen brain tissue and buccal cells	HM450K	Bonferroni corrected (did not control for BMI, smoking, etc.)	244 hypomethylated and 188 hypermethylated CpG sites in cases and significantly correlated between brain and buccal cells
Wang et al., 2016	16 male and seven female European–Australian pairs of AUD and control subjects	DSM‐IV	Prefrontal cortex	HM450K	FDR corrected, corrected for multiple testing; age, sex and BMI used as covariates.	1812 CpGs differentially methylated in males and none in females
Lohoff et al., 2018	Discovery: 23 AUD subjects (16 male and 7 female) and 23 age‐matched healthy controls; NICHD: *n* = 29 cases 29 controls with MDD; 68 AUD and 72 healthy controls	DSM‐IV	Blood and brain	HM450K	Discovery: controlled for age, sex and neuronal proportion NICHD: controlled for age, sex and postmortem interval and multiple testing using FDR; age, gender, AIM scores, and scanner types included as nuisance covariates	Five differentially methylated genes in brain after FDR correction
Gatta et al., 2019	25 AUD cases and 25 controls	DSM‐IV	Prefrontal cortex	HM450K	Benjamini‐Hochberg and FDR‐corrected	5254 differentially methylated genes in cases
Lohoff et al., 2020	Discovery: 336 AUD and 203 controls First RC: (43 AUD and 43 controls) Second RC: (4,301 Scottish) Third RC: (392 Grady Trauma Project) Postmortem Brain 1: 58 individuals with and without MDD Postmortem Brain 2: 23 participants with alcohol dependence/abuse and 23 age‐matched controls	Discovery: SCID‐IV First RC: SCID‐IV Second RC: Clinical Questionnaire Third RC: SCID‐IV Postmortem Brain 1: alcohol problem based on clinical interview Postmortem Brain 2: DSM‐IV	Discovery: blood First RC: blood Second RC: blood Third RC: blood Post‐Mortem Brain 1: neuronal and glial tissues Postmortem Brain 2: brain	Discovery: HM850K First RC: Infinium HM850K Second RC: HM850K Third RC: HM850K 450K Post‐Mortem Brain 1: 450K Post‐Mortem Brain 2: 450K	*M* values corrected for age, sex, smoking status, pack years of smoking, relatedness, batch and estimated cell counts.	4798 cites differentially methylated in AU

Abbreviations: AA: African–American; BMI: body mass index; CF: confounding factor; CIWA‐Ar: Clinical Institute Withdrawal Assessment for Alcohol (revised version); CVD: cardiovascular disease; DSM‐IV: Diagnostic and Statistical Manual of Mental Disorders, fourth edition; EA: European–American; FDR: False Discovery Rate; HM450K: Infinium HumanMethylation 450K BeadChip; HM850K: Infinium Methylation EPIC BeadChip; ND: nicotine dependence; NICHD: National Institute on Child Health and Human Development; SCID‐IV: Structured Clinical Interview for DSM‐IV; SSAGA‐II: Semi‐Structured Assessment for the Genetics of Alcoholism.

### 2.1 Candidate gene methylation studies

Candidate gene methylation studies are based upon a priori hypotheses that a disease is associated with variation of DNA methylation in the promoter region or around transcription start sites of a specific gene (supporting information Table S1). These studies primarily used pyrosequencing or bisulfite sequencing. Though bisulfite sequencing has been shown to be more sensitive than pyrosequencing, the two methods generally yield similar results.^21^ Although reviews investigating genetic studies of candidate genes have shown them to not be replicable, it has not been clear if that is also true for candidate gene methylation studies.^22^ In our review of candidate gene methylation studies of AUD, we also found conflicting results. Certain findings were replicated in subsequent EWAS. *Aldehyde Dehydrogenase 2 Family Member* (*ALDH2*)^23^ and *Opioid Receptor Mu 1 (OPRM1)* ^24^ were both found to be hypermethylated in candidate gene methylation studies and also significantly hypermethylated in EWAS of AUD.^25^, ^26^ *Orexin* ^27^ was neither shown to be differentially methylated in a candidate gene methylation study nor differentially methylated in any EWAS we reviewed. The majority of candidate gene methylation studies, however, were not replicated, with many contradicted by subsequent findings. For example, *Glutamate Ionotropic Receptor NMDA Type Subunit 2B* (*GRIN2B*),^25^, ^28^ *Ganglioside Induced Differentiation Associated Protein 1* (*GDAP1*),^29^, ^30^ *Somatostatin Receptor 4* (*SSTR4*),^31^, ^32^ and *Solute Carrier Family 6 Member 3* (*SLC6A3*)^26^, ^33^, ^34^, ^35^, ^36^ were all found to have conflicting results across studies.

### 2.2 EWAS

EWAS refer to studies that survey specific CpG sites across the genome without a priori hypotheses. Given the failures to replicate candidate gene methylation studies and the limitations they have in explaining AUD, there has been a recent emphasis on EWAS in better understanding DNA methylation in AUD. This has been made possible by the development of array‐based platforms that offer relatively easy, high through‐put methods for assessing CpG sites in nearly 99% of Reference Sequence database genes, with valid and standardized methods for processing, quality control, and analysis.^37^, ^38^, ^39^ The chips range in their coverage, from the first‐generation Illumina 27K introduced in 2008 to the Illumina 450K in 2011 and the EPIC Chip in 2016, which cover roughly 27,000, 450,000 and 850,000 CpG sites, respectively. Unlike Sanger sequencing of bisulfite‐converted DNA, which allows for assessment of DNA methylation across the entire genome, it is important to note that the Illumina arrays used in the studies we reviewed only survey a fraction of the genome with a bias toward CpG sites in promoter regions.^40^

EWAS can generate thousands of significant hits, so it is much more difficult to determine if they are replicable. Still, it is equally important to determine how reliable this mode of inquiry is. To assess whether there was overlap between significant CpG sites in EWAS, we aggregated all available CpG sites and identified which genes had significantly differential methylation in two or more studies. We found 180 CpG sites that were hits in two studies and four that were hits in three studies (*p* value < 0.05, Tables 1, 2 and S2). Although these replicable hits provide targets for molecular follow‐up experiments, it is important to note that this represents a small amount of overlap between significant CpGs, because several studies had over 1000 significant hits. Discrepancies in significant CpGs may be in part due to differences in tissue types as methylation signatures vary by cell type.^41^ Of the EWAS investigating AUD, seven were in blood, five were in brain, one was in liver, and one was in buccal cells with some using more specific components of blood or brain (Table 1). Other factors that may explain these discrepancies include differences in AUD diagnostic tools, differences in demographic characteristics of patient populations and small sample sizes.

**TABLE 2:** Genes near differentially methylated CpG sites in three or more studies

Gene	Tissue	Studies
*HNRNPA1*	Blood, lymphocytes, postmortem brain and saliva	Lohoff et al., 2020; Philibert et al., 2014; Witt et al., 2020
*LMF1*	Blood, brain and buccal	Hagerty et al., 2016; Lohoff et al., 2018; Witt et al., 2020
*LRRC20*	Blood, brain and buccal	Hagerty et al., 2016; Lohoff et al., 2018; Witt et al., 2020
*PLEKHG4B*	Blood, brain and buccal	Hagerty et al., 2016; Lohoff et al., 2018; Witt et al., 2020

## 3 RECENT MOLECULAR DISCOVERIES

Despite the low replicability across EWAS, several potential targets for further study emerged from our analysis. In particular, there were four genes that were shown to be differentially methylated in three different studies (Table 2). We searched major studies of alcohol consumption for all of the genes replicated in three AUD studies.^26^, ^42^, ^43^ *Heterogenous Nuclear Ribonucleoprotein A1* (*HNRNPA1*) was significantly hypomethylated in AUD cases in three studies that included saliva, lymphocytes and blood,^29^, ^44^, ^45^ and *HNRNPA1* methylation was inversely associated with alcohol consumption in blood^42^, ^46^ and saliva.^26^ HNRNPA1 regulates RNA processing, including transcription, translation, splicing, stability and export. It is also involved in microRNA processing, telomere maintenance and transcription factor activation.^47^ It has been shown to both activate and repress gene expression. For example, it transactivates *Apolipoprotein E* (*APOE)*, which is associated with the metabolism of fats and Alzheimer's disease.^48^ HNRNPA1 has also been shown to play a role in the degradation of IKBa, an inhibitor of *Nuclear Factor Kappa‐B* (*NF‐κB*) transcription. Cells without *HNRNPA1* have been found to be unable to synthesize NF‐*κ*B,^49^ and increases in NF‐*κ*B have been shown to be associated with neuroinflammation, chronic alcohol consumption and alcohol‐induced hepatic inflammation.^50^, ^51^, ^52^ Epigenetic modifications in *HNRNPA1* may help mediate this relationship between NF‐*κ*B and AUD, as well as explain some of the transcriptional differences associated with alcohol consumption.^53^ *Lipase maturation factor 1* (*LMF1*) was also differentially methylated in three studies of AUD in blood, brain and buccal cells^44^, ^54^, ^55^ along with one study of alcohol consumption in blood.^46^ LMF1 is a chaperone that activates vascular lipases necessary for lipid clearance, and thus, mutations have been associated with hypercholesteremia.^56^, ^57^

Little is known about the other two most common differentially methylated genes. *Leucine rich repeat containing 20* (*LRRC20*) was differentially methylated in both studies of AUD^44^, ^54^, ^55^ and a study of alcohol consumption.^46^ It has been associated with autoantibody‐independent changes in interferon‐alpha levels, and leucine‐rich repeats have been associated with immune responses, but the function of this protein is far from understood.^58^ Finally, *pleckstrin homology and RhoGEF domain containing GHB* (*PLEKHG4B*) is also poorly understood. Variants appear to be associated with white and gray matter diffusivity in individuals with diabetes mellitus,^59^ but little else is known about the protein. Although it can be difficult to infer much from genes that are poorly understood, a theme—namely, that of immune function—does emerge. Though lipid regulation by LMF1 may seem distinct, lipids have been associated with neuroimmune responses and neurodegeneration.^60^ They have even been shown to play a causal role in pro‐inflammatory microglial states associated with aging.^61^

## 4 BEYOND MOLECULES: RELEVANT MOLECULAR PATHWAYS

We found that genes involved in immune responses and regulation were also commonly enriched in gene ontology term and pathway analyses of AUD. Analyzing gene ontology terms that classify genes based on their relevance to functional categories and biological pathways can be more useful than identifying single genes, as biological systems are complex and contain many redundancies—altering any one of the steps in a pathway could lead to a pathological cascade, the relevance of which may not be apparent when looking at individual modifications in gene expression. One difficulty in assessing whether pathways are replicable is that there are different libraries, including GoMiner, Kyoto Encyclopedia of Genes and Genomes (KEGG), and WikiPathways. Nevertheless, our review found that there were 15 pathways that were significant in at least two studies and three that were significant in three studies (Table 3). Aside from pathways related to immune regulation, there are several related to basic biological processes, such as signaling, that are involved in immune responses as well as many other cellular functions.

**TABLE 3:** Pathways significant in multiple studies

Pathway	Studies
Autoimmune thyroid disease	Zhang et al., 2013; Zhao et al., 2013
Cell adhesion	Zhao et al., 2013; Witt et al., 2020
Cell part morphogenesis	Philibert et al., 2014; Wang et al., 2016
Establishment of localization	Lohoff et al., 2020; Witt et al., 2020
Defense response to bacterium	Gatta et al., 2019; Witt et al., 2020
Immune response	Gatta et al., 2019; Witt et al., 2020
Immune system process	Philibert et al., 2014; Zhang et al., 2013; Witt et al., 2020
Intracellular signal transduction	Philibert et al., 2014; Witt et al., 2020
Integral to membrane	Gatta et al., 2019; Witt et al., 2020
Protein binding	Gatta et al., 2019; Philibert et al., 2014
Response to external stimulus	Gatta et al., 2019; Lohoff et al., 2020; Zhang et al., 2013
Response to stimulus	Witt et al., 2020; Zhang et al., 2013
Response to stress	Gatta et al., 2019; Lohoff et al., 2020; Zhang et al., 2013
Signaling	Philibert et al., 2014; Witt et al., 2020
Transport	Lohoff et al., 2020; Witt et al., 2020

The finding that immune system processes emerge across molecular and pathway analyses provides an interesting human parallel to research into the role of the immune system in rodent models of AUD. As most of the studies in this review with meaningfully large sample sizes were in blood, however, these results should be interpreted with caution. DNA in blood is extracted from leukocytes, so there is a bias in our results toward sampling immune cells for methylation analyses in blood. Still, the importance of immune pathways was relevant across tissue types. Like epigenetics, the immune system represents an interface between the genome and the environment and thus may also integrate genetic variants with environmental signals to affect neurobiological processes.^62^

Though far from painting a full picture of AUD pathology, animal studies have found alcohol to cause neuroinflammation, which has in turn been associated with AUD‐related psychiatric symptoms. In rodents, chronic, heavy alcohol exposure has been shown to lead to alcohol dependence and voluntary self‐administration, which suggests that understanding the physiological effects of ethanol may lead to a better understanding of AUD psychopathology. In parallel, these models have shown neuroinflammation to develop as a result of ethanol exposure, and neuroinflammation has been independently associated with symptoms of psychiatric disorders often comorbid with AUD such as depression.^63^, ^64^ Evidence suggesting that the inflammatory response may play a causal role in AUD pathology has emerged as researchers have found administration of anti‐inflammatory agents reduces both neuroinflammation and alcohol administration and escalation in alcohol‐dependent rodents.^65^, ^66^ These findings are far from conclusive because there have been only a handful of studies showing that reductions in neuroinflammation reduce relapse‐like behaviors and replicability has long been an issue with animal models of disease. In addition, the anti‐inflammatory agents used are not fully understood and may be affecting alcohol behaviors through a pathway other than neuroinflammation. Still, these findings in animal models, in combination with our findings that DNA methylation is consistently altered in both immunological genes and pathways in AUD, highlight the promise of studying neuroimmunology in the context of alcohol‐related pathology.

## 5 DISCUSSION

We found three primary categories of studies investigating DNA methylation in AUD. The first category measured global methylation in AUD, but all global methylation studies were small with substantially low power to detect the variation. Because global levels of DNA methylation generally do not appear to be very informative or useful in detecting AUD or understanding its etiology, we excluded them in the analysis. We identified candidate gene methylation studies, where genes were studied based on a priori hypotheses, as the second category. Most candidate gene methylation studies were not replicable, with the exception of *ALDH2* and *OPRM1*. The third category, EWAS, was used to survey the genome in individuals with AUD with no a priori hypotheses. The most replicated findings among EWAS were of *HNRNPA1*, *LRRC20*, *PLEKHG4B*, and *LMF1* in AUD. HNRNPA1, LRRC20 and LMF1 all appear to play some role in immune regulation, which was also repeatedly found to be differentially methylated in pathway analyses of EWAS data. However, it should be noted that these genes are just the first surfacing from initial EWAS studies, and likely there will be multiple others, as the epigenetic architecture of AUD is complex.

## 6 LIMITATIONS AND FUTURE DIRECTIONS

Moving forward, there are several limitations to the studies we reviewed that may have straightforward solutions. For example, most of the studies suffer from small sample sizes, which may explain the lack of replicability across studies. There are also differences in tissue type, diagnostic criteria, preprocessing, and array technology, which precluded meta‐analysis in this instance. In addition, statistical methods varied widely across studies, with many not controlling for smoking, which is often comorbid with AUD and which also has strong epigenetic effects.^67^ Studies also varied on their significance thresholds, as well as whether they used standard corrections for cell type proportion, multiple testing, batch effects and false discovery rates. If standardized procedures are developed for quality control, normalization, exclusion of low‐quality samples and methylation outliers, and statistical controls for confounding variables, the limits of small sample sizes may be overcome with valid and comprehensive meta‐analyses.

Other barriers will require more profound advances to overcome. A significant barrier to understanding DNA methylation in AUD involves the nature of the epigenome. Unlike the genome, the majority of DNA methylation profiles are temporally dynamic and can vary significantly by cell type, meaning bulk tissue analyses may miss important signals from specific cells.^68^ Because DNA methylation varies temporally, cross‐sectional epigenetic studies cannot differentiate between cause and effect. This issue is of particular relevance to substance use disorders like AUD, where the disorder is associated with repeated use of a substance with demonstrated pharmacological and epigenetic effects. Witt et al.^44^ examined changes in DNA methylation in men during 2 weeks of withdrawal and found that this period of abstinence reduced the differences in methylation sites between AUD cases and controls from 9845 to 6094. As recent drinking patterns can vary widely within AUD cases and non‐AUD controls, these patterns should be taken into consideration when assessing DNA methylation signatures.

More broadly, given that alcohol consumption is a core component of AUD, it will be important to distinguish epigenetic signatures that may predispose individuals to AUD from those that may result from alcohol consumption. As Witt et al. demonstrated, many of the AUD‐associated DNA methylation sites in peripheral tissues were reversed after a short period of abstinence, which suggests many sites identified in studies of AUD are likely associated with alcohol consumption rather than the etiology of AUD. This is important because genetic studies of AUD have found the genetic variants associated with AUD and alcohol consumption to be distinct.^69^ More specifically, genetic variants associated with AUD appear to be more closely related to variants associated with psychiatric disorders, while those associated with alcohol consumption appear to be more closely associated with metabolism.^69^ Because data on alcohol consumption are much easier to collect, studies of DNA methylation in alcohol consumption have, on the whole, been much larger than those in AUD. For example, Liu et al.^42^ looked at alcohol consumption using a sample size (*n* = 13,317) much larger than all of the AUD studies combined. Dugue et al.,^46^ Wilson et al.,^43^ and Xu et al.^26^ also had large sample sizes. It is interesting that not all of the genes that were significant in two or more AUD studies overlapped with larger studies of alcohol consumption, and future studies investigating AUD in larger sample sizes could help determine if there are different epigenetic themes characteristic of AUD versus alcohol consumption. Additionally, prospective studies that track patients for longer periods of abstinence may help to distinguish between CpG sites that may be associated with the psychopathology of AUD and those that are a consequence of alcohol consumption.

Another important consideration when assessing AUD and DNA methylation is the high rate of comorbidities among psychiatric disorders. AUD frequently co‐occurs with many psychiatric disorders, including other substance use disorders, major depressive disorder, anxiety disorders, personality disorders and psychotic disorders among others.^70^ The high incidence of psychiatric comorbidities could exist for many reasons. AUD may cause other psychiatric symptoms, or psychiatric symptoms may cause symptoms of AUD. These psychiatric disorders could also share an underlying pathology. Most likely, the high correlations exist due to some combination of these three explanations.

This clinical heterogeneity and diagnostic uncertainty persist when assessing the DNA methylation differences associated with AUD. It is not only unclear whether DNA methylation signatures are a cause or consequence of AUD but also if they are a cause or consequence of the many psychiatric comorbidities associated with AUD. Some studies account for this by excluding individuals with specific psychiatric comorbidities or by controlling for variables like smoking in their statistical analyses. A different approach is to instead look at epigenetic signatures associated with AUD and specific comorbidities, for example, to look at those with comorbid AUD and post‐traumatic stress disorder (PTSD). This may have more clinical utility given that these disorders often present together. Xu et al.^25^ (2017), for example, looked at alcohol and nicotine codependence, and Xu et al.^26^ (2019) looked at alcohol consumption in veterans, a unique population with higher incidences of disorders like PTSD. Studying DNA methylation changes associated with more specific phenotypes of AUD such as AUD with specific comorbidities or with specific drinking patterns may help us to understand these different phenotypes as well as the variability in DNA methylation signatures associated with AUD.

Another limitation related to AUD and other psychiatric disorders is that most DNA methylation studies are limited to blood, saliva, or buccal cell sources. Studies in both postmortem and living human brain tissue have found limited correlations between methylation patterns in the brain and peripheral tissue of patients, with the highest interindividual correlation between nominally significant CpG sites in brain and blood at roughly 20%.^71^, ^72^, ^73^ However, some analyses have shown that the correlation between methylation across brain and blood is stronger at certain CpG sites, and analyses of DNA methylation in blood could focus on these sites.^72^ Studies of methylation in brain tissue from AUD cases rely on postmortem cohorts, which usually have a limited number of subjects. The effects of variables such as postmortem delay, or death itself, on methylation are unclear. In addition, AUD is dynamic across the lifespan and often goes undiagnosed, which poses challenges with correct classification of cases and controls.

Despite these limitations, there are several future directions that emerge. First, the most comprehensive of the arrays used to assess the genome, the Infinium MethylationEPIC BeadChip, still only covers roughly 850,000 of 28 million CpG sites. Whole genome bisulfite sequencing (WGBS) can measure DNA methylation at nearly every CpG site in the genome.^38^ Though WGBS requires more DNA and is more labor intensive and expensive than methylation arrays, it is widely used in other fields and may expose methylation patterns important to AUD that are not captured in the methylation arrays used to date. A more realistic intermediate approach may be targeted DNA methylation capture sequencing, which involves bisulfite sequencing at targeted regions of the genome. Targeted capture sequencing technologies cover roughly 10%–13% of the methylome, which is significantly greater than the 3% of CpG sites covered by the EPIC chip. Some technologies also allow for customization of the region surveyed, which may be useful in assessing hypothesized regions of interest.

Going forward, it will also be important to understand the functional relevance of the molecular targets identified in these studies. This will require integration of genetic, epigenetic and gene expression data. One strategy to better investigate the relationship between genetic variants and epigenetic changes is to incorporate methylation quantitative trait loci (mQTLs), which are genetic variants that may affect DNA methylation patterns. EWAS could integrate genetic data to determine if mQTLs, or genetic variants associated with DNA methylation in specific regions, are related to DNA methylation patterns in AUD. Metastable epialleles, which are alleles that are particularly vulnerable to environmental exposures and show differential expression in genetically identical individuals, could also be explored. It will also be important to better understand how DNA methylation relates to gene expression. DNA methylation has traditionally been thought to repress transcription. However, increasing evidence shows that its role may be more complicated and it will be important to not only identify DNA methylation but also begin to understand how this methylation affects transcription. This will also require distinguishing between methylation and hydroxymethylation, because hydroxymethylation may be associated with demethylation or have a different effect on gene expression than methylation at any given site. Although existing bisulfite sequencing methods do not distinguish between the two, oxidative bisulfite sequencing can be used to remove hydroxymethylated sites in order to assay methylation alone. The methylation results can then be compared to traditional bisulfite sequencing to discern levels of hydroxymethylation. Though more labor intensive, use of both bisulfite and oxidative bisulfite sequencing in AUD may help to distinguish between these two types of methylation.

In addition to understanding how DNA methylation affects gene transcription, we must understand the temporal order of methylation and functional significance of affected proteins to understand its biological effects. Animal models of AUD or AUD‐related symptoms such as compulsive alcohol administration can provide a system in which to interrogate some of these questions, like what methylation signatures are associated with the development of measurable AUD symptoms such as self‐administration of alcohol, tolerance, or withdrawal, or what modifications result from chronic, heavy alcohol exposure. There have been several instances where methylation has provided insight and therapeutic targets for AUD. For example, DNA methyltransferase inhibitors have been shown to reduce alcohol consumption in rodents, though further replication is needed.^70^ A better understanding of this process may provide insight into why different methylation patterns are present in AUD and how that impacts disease progression. In addition, genetic targets that are differentially methylated may provide insight into disease processes. For example, proprotein convertase subtilisin‐kexin 9 (PCSK9), which is primarily expressed in the liver, was first identified in relation to AUD when it was differentially methylated in an EWAS.^55^ A follow‐up study found that inhibition of PCSK9 by alirocumab, a monoclonal antibody recently approved by the Food and Drug Administration for the treatment of familial hypercholesteremia, attenuated alcoholic liver disease progression, demonstrating the promise of exploring the targets identified in this review in vivo.^74^

Another potential application of EWAS is the development of biomarkers for AUD. Studies and treatment of AUD are currently limited by diagnostic interviews, for which there is moderate‐to‐high reliability between raters.^75^ In addition to the potential variability introduced by different raters, diagnoses are ultimately based upon an individual's response to criteria, which may be impacted by social desirability bias and imperfect recall. In this context, a biomarker of alcohol‐related phenotypes may improve the reliability of alcohol‐related classifications in research and clinical care. Biomarkers have already shown promise in assessing alcohol consumption—Liu et al.^42^ used EWAS data to identify 144 CpG sites that predicted heavy alcohol consumption with an area under the curve (AUC) of 0.90–0.99, where AUC represents a measure of biomarker accuracy on a scale from 0 to 1, with 1 being the most accurate. Liang et al.^76^ also found methylation to have potential as a biomarker. They identified 143 CpG sites that predicted phosphatidylethanol (PeTH), a phospholipid metabolite shown to persist in blood for up to 3 weeks after alcohol consumption, with an AUC of 0.9 in a training dataset and an AUC of 0.8 in a replication dataset.^76^ The studies had few overlapping predictors and both require further validation. It also remains unclear whether epigenetic biomarkers would be stable over longer periods of time or have higher resolution than PeTH. Still, biomarkers of alcohol consumption are a promising area of research.

Recently, methylation risk scores, the DNA methylation equivalent to polygenic risk scores, have emerged as a specific type of biomarker. With regard to alcohol consumption, methylation risk scores have been shown to be much better at predicting consumption than polygenic risk scores, explaining roughly 12.5% of phenotypic variance compared to polygenic risk scores, which explain roughly 0.7% of variance.^77^ Larger EWAS may enable the development of AUD biomarkers and methylation risk scores. Biomarkers of AUD may help clinicians identify AUD in patients so they can better implement appropriate interventions. In research, biomarkers could lead to more objective classifications of AUD, which would in turn improve the validity of studies. In addition, biomarkers of AUD and alcohol consumption could be used in tandem to better address patients' needs and to better parse alcohol consumption and AUD psychopathology in research studies.

## 7 CONCLUSIONS

The assessment of DNA methylation in AUD is a rapidly evolving and promising new area of research. In our review, we identified 184 genes that were consistently differentially methylated in at least two EWAS, with four of them, *HNRNPA1*, *LMF1*, *LRRC20* and *PLEKHG4B*, replicated in three EWAS. In addition, we compared gene ontological analyses and found 15 pathways overlapping between studies, the most common relating to immune regulation and basic cellular processes. Though differences in diagnostic criteria, methylation assays and preprocessing precluded a meta‐analysis in this instance, our analysis identified promising targets for future epigenetic and genetic studies of AUD and provided an update on where the field has come and important questions that must be resolved in the future. The biggest challenge going forward with EWAS will be identifying the functional significance of DNA methylation patterns and parsing the epigenetic signatures that may predispose individuals to AUD from those that result from confounding variables such as smoking or from alcohol consumption itself. The most immediate application of DNA methylation data appears to be in studying the function and therapeutic relevance of the genes that are differentially methylated across studies and in the development of biomarkers of AUD that may yield more accurate tools for clinical and research‐related diagnostic purposes.

## AUTHOR CONTRIBUTIONS

MJL and FWL were responsible for the conception of the review. MJL and JL performed the literature search. MJL, JL, JJ, and FWL wrote the manuscript. All authors critically reviewed content and approved the final version for publication.

## Supporting information


**Table S1.** Candidate Gene Methylation Studies of Alcohol‐Related Phenotypes
**Table S2.** Genes near differentially methylated CpG sites in two studies
**Table S3.** Similar Pathways Differentially Methylated Across Studies
