# Supplementary material for: Epigenetics of alcohol use disorder—A review of recent advances in DNA methylation profiling
Source: Addict Biol. 2021 Feb 3;26(6):e13006. doi: 10.1111/adb.13006 (PMC8596445; doi:10.1111/adb.13006)
Supplement: Supplementary file 1 — Table S1. Candidate Gene Methylation Studies of Alcohol‐Related Phenotypes Table S2. Genes near differentially methylated CpG sites in two studies Table S3. Similar Pathways Differentially Methylated Across Studies [file ADB-26-e13006-s001.docx]

**Supplementary Table 1. Candidate Gene Methylation Studies of Alcohol-Related Phenotypes**

| **Author** | **Sample** | **Diagnosis** | **Tissue** | **DNA Methylation Assay** | **Results** |
| --- | --- | --- | --- | --- | --- |
| **Glahn et al. 2014** | 99 AUD and 99 age and BMI matched controls | ICD-10/ DSM-IV | Blood | Bisulfite sequencing | Decreased *ANP* promoter methylation in cases, no difference in *AVP* promoter methylation between cases and controls |
| **Muschler et al. 2014** | 84 men with AD | DSM-IV, OCDS for alcohol craving | Blood | Bisulfite sequencing | No differences in *POMC* methylation associated with alcohol craving (p=0.023) |
| **Heberlein et al. 2015** | 99 AD and age matched healthy controls | ICD-10 and DSM-IV | Blood | Sodium bisulfite | Mean methylation of the *BDNF* promoter significantly increased in AD patients compared to healthy controls (p<0.001) and decreased during alcohol withdrawal; mean BDNF promoter methylation significantly higher in group who abstained longer before relapse |
| **Bruckmann et al. 2016** | 49 AUD and 37 controls | AUDIT and GSI | Blood | Pyrosequencing | *GDAP1* hypomethylated in AUD, which is reversed during short-term alcohol treatment |
| **Haschemi et al. 2016** | 82 with alcohol dependence and 34 matched controls | DSM-IV and ICD-10 | Blood | Bisulfite sequencing | *ALDH2* methylation higher in AD cases on day 1 but not day 7 or day 14 of treatment |
| **Berent et al. 2017** | 176 patients with AD/127 healthy controls | ICD-10/AUDIT | Buccal cells | Bisulfite sequencing | *SSRT4* not differentially methylated in AUD cases |
| **Gatta et al. 2017** | 25 AD /25 controls | DSM-IV | Post-mortem brain | Immunoprecipitation | Increased methylation in the promoter region of the delta subunit of the GABA_A_ receptor in AD cases |
| **Muench et al. 2017** | 45 AUD/45 healthy controls | DSM-IV | Blood | Bisulfite pyrosequencing | Decreased methylation of *SLC6A3* predicted nucleus accumbens activation during high loss anticipation but not in individuals with AUD |
| **Checknita et al. 2018** | 114 women recruited for substance misuse treatment/23 controls | DSM-IV/AUDIT | Saliva | Bisulfite sequencing | No significant association between *MAOA* methylation and AUD status |
| **Gangisetty et al. 2019** | 33 moderate drinkers, 6 binge drinkers and 8 heavy drinkers with no diagnosis of alcohol dependence | DSM-IV | Blood | Methylation-specific PCR | *PER2* and *POMC* methylation increased in moderate to heavy drinkers, and positive associated with subjective alcohol cravings and amount of alcohol consumed during alcohol taste test |
| **Muench et al. 2019** | 45 individuals with AD/45 controls | DSM-IV | Blood | Pyrosequencing | No difference in *SLC6A4* promoter methylation in cases versus controls |

ICD-10: International Statistical Classification of Diseases, Tenth Revision; DSM-IV: Diagnostic and Statistical Manuel of Mental Disorders, Fourth Edition; ANP: atrial natriuretic peptide; AVP: arginine vasopressin; AD: alcohol dependence; OCDS: Obsessive Compulsive Drinking Scale; POMC: proopiomelanocortin; BDNF: brain-derived neurotrophic factor; GSI: gambling severity index; GDAP1: ganglioside induced differentiation associated protein 1; ALDH2: aldehyde dehydrogenase 2 family member; SLC: solute carrier; AUDIT: Alcohol Use Disorders Identification Test; SSRT4: somatostatin receptor subtype-4; GABA: gamma aminobutyric acid A; SLC6A3: solute carrier family 6 member 3; MAOA: monoamine oxidase A; ADS: Alcohol Dependence Scale; ICS: Impaired Control Scale; DRD2: dopamine receptor D2; PER2: period circadian regulator 2; SLC6A4: solute carrier family 6 member 4; RT-PCR: real-time polymerase chain reaction

**Supplementary Table 2. Genes near differentially methylated CpG sites in two studies**

| **Gene** | **Tissue** | **Studies** |
| --- | --- | --- |
| *ABCB8* | blood & bulk brain | Lohoff et al. 2018, Witt et al. 2020 |
| *ABLIM3* | blood, brain and buccal | Hagerty et al. 2016, Witt et al. 2020 |
| *ABR* | blood, brain, buccal and lymphoblasts | Hagerty et al. 2016, Witt et al. 2020 |
| *ACSF3* | brain and buccal | Hagerty et al. 2016, Witt et al. 2020 |
| *ADAM19* | blood & bulk brain | Lohoff et al. 2018, Witt et al. 2020 |
| *AMBRA1* | blood | Lohoff et al. 2020, Wilt et al. 2020 |
| *AMZ1* | brain and buccal | Hagerty et al. 2016, Witt et al. 2020 |
| *ANKK1* | blood | Witt et al. 2020, Xu et al. 2017 |
| *AP2A2* | brain and buccal | Hagerty et al. 2016, Witt et al. 2020 |
| *ARHGAP22* | brain and buccal | Hagerty et al. 2016, Witt et al. 2020 |
| *ARID3A* | brain and buccal | Hagerty et al. 2016, Witt et al. 2020 |
| *ARRB1* | blood | Lohoff et al. 2020, Witt et al. 2020 |
| *ARRB1* | blood | Lohoff et al. 2020, Witt et al. 2020 |
| *ASAP2* | brain and buccal | Hagerty et al. 2016, Witt et al. 2020 |
| *ATP6V1B2* | blood | Lohoff et al. 2020, Wilt et al. 2020 |
| *ATXN7L1* | brain and buccal | Hagerty et al. 2016, Witt et al. 2020 |
| *B2M* | blood | Lohoff et al. 2020, Wilt et al. 2020 |
| *BAHD1* | blood, brain and buccal cells | Hagerty et al. 2016, Witt et al. 2020 |
| *BNIPL* | blood | Lohoff et al. 2020, Wilt et al. 2020 |
| *BOC* | blood, brain and buccal | Hagerty et al. 2016, Witt et al. 2020 |
| *C14orf132* | blood, brain and buccal | Hagerty et al. 2016, Witt et al. 2020 |
| *C1orf109* | blood, brain and buccal | Hagerty et al. 2016, Lohoff et al. 2018 |
| *C5orf38* | blood, brain and buccal | Hagerty et al. 2016, Witt et al. 2020 |
| *CABLES1* | blood, brain | Lohoff et al. 2020, Wilt et al. 2020 |
| *CAMK2G* | blood | Lohoff et al. 2020, Wilt et al. 2020 |
| *CCDC57* | blood, brain and buccal | Hagerty et al. 2016, Witt et al. 2020 |
| *CCDC85C* | blood | Witt et al. 2020, Zhao et al. 2015 |
| *CCND1* | blood, brain and buccal | Hagerty et al. 2016, Witt et al. 2020 |
| *CDH4* | blood, brain and buccal | Hagerty et al. 2016, Witt et al. 2020 |
| *CHRM2* | blood | Witt et al. 2020, Xu et al. 2017 |
| *COL22A1* | blood & bulk brain | Lohoff et al. 2018, Witt et al. 2020 |
| *CPLX2* | PFC, blood | Wang et al. 2015, Witt et al. 2020 |
| *CREB1* | blood | Witt et al. 2020, Xu et al. 2017 |
| *CYB561* | brain and buccal | Hagerty et al. 2016, Witt et al. 2020 |
| *DBH* | blood, brain and buccal | Hagerty et al. 2016, Xu et al. 2017 |
| *DDC* | blood | Witt et al. 2020, Xu et al. 2017 |
| *DGKZ* | blood, brain and buccal | Hagerty et al. 2016, Witt et al. 2020 |
| *DHX9* | blood, brain and buccal | Hagerty et al. 2016, Witt et al. 2020 |
| *DNAH10* | blood | Lohoff et al. 2020, Wilt et al. 2020 |
| *DNMT1* | blood | Witt et al. 2020, Xu et al. 2017 |
| *DNMT3A* | Blood | Witt et al. 2020, Xu et al. 2017 |
| *DNMT3B* | Blood | Witt et al. 2020, Xu et al. 2017 |
| *DOCK5* | blood, brain and buccal | Hagerty et al. 2016, Witt et al. 2020 |
| *DPF3* | blood, brain and buccal | Hagerty et al. 2016, Witt et al. 2020 |
| *DRD4* | blood, brain and buccal | Hagerty et al. 2016, Xu et al. 2017 |
| *ELMSAN1* | blood | Lohoff et al. 2020, Witt et al. 2020 |
| *ELOVL5* | blood, brain and buccal | Hagerty et al. 2016, Witt et al. 2020 |
| *EPHA10* | blood, brain and buccal | Hagerty et al. 2016, Witt et al. 2020 |
| *EXD3* | brain and buccal | Hagerty et al. 2016, Witt et al. 2020 |
| *FADS3* | blood, brain and buccal | Hagerty et al. 2016, Witt et al. 2020 |
| *FANCA* | brain and buccal | Hagerty et al. 2016, Witt et al. 2020 |
| *FBXO17* | blood & bulk brain | Lohoff et al. 2018, Witt et al. 2020 |
| *FHIT* | blood, brain and buccal | Hagerty et al. 2016, Witt et al. 2020 |
| *FKBP5* | blood, post-mortem brain | Lohoff et al. 2020, Witt et al. 2020 |
| *FOXP1* | blood | Lohoff et al. 2020, Witt et al. 2020 |
| *FSHB* | blood & bulk brain | Lohoff et al. 2018, Witt et al. 2020 |
| *GABRB3* | blood, brain and buccal | Hagerty et al. 2016, Xu et al. 2017 |
| *GAS5* | blood, post-mortem brain | Lohoff et al. 2020, Witt et al. 2020 |
| *GFI1* | blood, lymphocytes | Philibert et al. 2014, Witt et al. 2020 |
| *GLTSCR1* | blood, post-mortem brain | Lohoff et al. 2020, Wilt et al. 2020 |
| *GRAMD4* | blood, brain and buccal | Hagerty et al. 2016, Witt et al. 2020 |
| *HAHRR* | Blood, brain, buccal cells | Hagerty et al. 2016, Lohoff et al. 2020 |
| *HDAC4* | blood & bulk brain | Lohoff et al. 2018, Witt et al. 2020 |
| *HECW2* | blood, post-mortem brain | Lohoff et al. 2020, Wilt et al. 2020 |
| *HIPK2* | blood, brain and buccal | Hagerty et al. 2016, Witt et al. 2020 |
| *HOXB3* | blood | Lohoff et al. 2020, Wilt et al. 2020 |
| *HPS4* | blood | Lohoff et al. 2020, Wilt et al. 2020 |
| *HS6ST1* | blood, brain and buccal | Hagerty et al. 2016, Witt et al. 2020 |
| *HSPG2* | blood, brain and buccal | Hagerty et al. 2016, Witt et al. 2020 |
| *HTR1B* | blood | Witt et al. 2020, Xu et al. 2017 |
| *IL1RAP* | blood | Lohoff et al. 2020, Wilt et al. 2020 |
| *INPP5A* | Blood, brain and buccal | Hagerty et al. 2016, Witt et al. 2020 |
| *INS-IGF2* | blood, brain and buccal | Hagerty et al. 2016, Witt et al. 2020 |
| *IRF8* | brain and buccal | Hagerty et al. 2016, Witt et al. 2020 |
| *KIAA0556* | blood, brain and buccal | Hagerty et al. 2016, Witt et al. 2020 |
| *KIAA1804* | blood, brain and buccal | Hagerty et al. 2016, Lohoff et al. 2018 |
| *LDLRAD4* | blood | Lohoff et al. 2020, Wilt et al. 2020 |
| *LINC01126* | blood | Lohoff et al. 2020, Wilt et al. 2020 |
| *LINC01599* | blood | Lohoff et al. 2020, Wilt et al. 2020 |
| *LINC-PINT* | blood | Lohoff et al. 2020, Wilt et al. 2020 |
| *LMTK2* | blood & bulk brain | Lohoff et al. 2018, Witt et al. 2020 |
| *LOC101927588* | blood | Lohoff et al. 2020, Wilt et al. 2020 |
| *LOC115110* | blood & bulk brain | Lohoff et al. 2018, Witt et al. 2020 |
| *LRAT* | blood, brain and buccal | Hagerty et al. 2016, Witt et al. 2020 |
| *LRP5* | Blood, prefrontal cortex | Wang et al. 2015, Witt et al. 2020 |
| *LRRN4* | blood, brain and buccal | Hagerty et al. 2016, Witt et al. 2020 |
| *MACC1* | blood, brain and buccal | Hagerty et al. 2016, Witt et al. 2020 |
| *MAD1L1* | blood, brain and buccal | Hagerty et al. 2016, Witt et al. 2020 |
| *MAGI1* | blood & bulk brain | Lohoff et al. 2018, Witt et al. 2020 |
| *MAPK1* | blood | Witt et al. 2020, Xu et al. 2017 |
| *MAPT* | brain and buccal | Hagerty et al. 2016, Witt et al. 2020 |
| *MBD3* | blood | Witt et al. 2020, Xu et al. 2017 |
| *MEF2C* | blood | Lohoff et al. 2020, Wilt et al. 2020 |
| *MGC15885* | blood, brain and buccal | Hagerty et al. 2016, Witt et al. 2020 |
| *MICAL3* | blood, brain and buccal | Hagerty et al. 2016, Witt et al. 2020 |
| *MPRIP* | blood & bulk brain | Lohoff et al. 2018, Witt et al. 2020 |
| *MSRA* | Blood, brain and buccal | Hagerty et al. 2016, Witt et al. 2020 |
| *MXRA7* | blood, brain and buccal | Hagerty et al. 2016, Witt et al. 2020 |
| *MYH10* | blood, post-mortem brain | Lohoff et al. 2020, Wilt et al. 2020 |
| *MYH9* | blood | Lohoff et al. 2020, Wilt et al. 2020 |
| *MYT1L* | blood, brain and buccal | Hagerty et al. 2016, Witt et al. 2020 |
| *NCAM1* | blood | Witt et al. 2020, Xu et al. 2017 |
| *NCOA5* | blood, brain and buccal | Hagerty et al. 2016, Witt et al. 2020 |
| *NFATC1* | brain and buccal | Hagerty et al. 2016, Witt et al. 2020 |
| *NFATC2* | blood | Lohoff et al. 2020, Wilt et al. 2020 |
| *NFIC* | Blood, prefrontal cortex | Wang et al. 2016, Witt et al. 2020 |
| *NOP56* | blood | Lohoff et al. 2020, Wilt et al. 2020 |
| *NOTCH1* | blood, post-mortem brain | Lohoff et al. 2020, Wilt et al. 2020 |
| *OBSCN* | blood & bulk brain | Lohoff et al. 2018, Witt et al. 2020 |
| *PCSK5* | blood & bulk brain | Lohoff et al. 2018, Witt et al. 2020 |
| *PCSK9* | blood, buccal and bulk brain | Hagerty et al. 2016, Lohoff et al. 2018 |
| *PDGFA* | blood, brain and buccal | Hagerty et al. 2016, Witt et al. 2020 |
| *PEX14* | Blood, post-mortem brain | Lohoff et al. 2020, Witt et al. 2020 |
| *PIK35R* | Blood | Lohoff et al. 2020, Witt et al. 2020 |
| *PIP5K1C* | blood & bulk brain | Lohoff et al. 2018, Witt et al. 2020 |
| *PKHD1* | blood, brain and buccal | Hagerty et al. 2016, Witt et al. 2020 |
| *PLD2* | blood | Lohoff et al. 2020, Wilt et al. 2020 |
| *PLXNC1* | blood, brain and buccal | Hagerty et al. 2016, Witt et al. 2020 |
| *PNOC* | blood | Witt et al. 2020, Xu et al. 2017 |
| *POU2F2* | blood, post-mortem brain | Lohoff et al. 2020, Wilt et al. 2020 |
| *PPIF* | blood, lymphocytes | Philibert et al. 2014, Witt et al. 2020 |
| *PPP1R16B* | blood, brain and buccal | Hagerty et al. 2016, Witt et al. 2020 |
| *PRKCZ* | blood, post-mortem brain | Lohoff et al. 2020, Witt et al. 2020 |
| *PROX1-AS1* | blood, brain and buccal | Hagerty et al. 2016, Witt et al. 2020 |
| *PRSS23* | blood, brain and buccal | Hagerty et al. 2016, Lohoff et al. 2020 |
| *PSMD13* | blood, brain and buccal | Hagerty et al. 2016, Witt et al. 2020 |
| *PTK2* | Blood, lymphocytes | Philibert et al. 2014, Witt et al. 2020 |
| *PTPN5* | blood | Witt et al. 2020, Xu et al. 2017 |
| *RAB20* | blood, brain and buccal | Hagerty et al. 2016, Witt et al. 2020 |
| *RABGEF1* | blood, PFC | Wang et al. 2015, Witt et al. 2020 |
| *RARA* | Blood | Lohoff et al. 2020, Witt et al. 2020 |
| *RASA3* | blood, brain and buccal | Hagerty et al. 2016, Witt et al. 2020 |
| *RASGRP1* | blood, post-mortem brain | Lohoff et al. 2020, Wilt et al. 2020 |
| *REEP3* | blood, brain and buccal | Hagerty et al. 2016, Witt et al. 2020 |
| *REL* | blood, brain and buccal | Hagerty et al. 2016, Witt et al. 2020 |
| *RFTN1* | Blood, brain and buccal | Hagerty et al. 2016, Witt et al. 2020 |
| *RIN2A* | blood | Witt et al. 2020, Xu et al. 2017 |
| *RP1L1* | blood, brain and buccal | Hagerty et al. 2016, Witt et al. 2020 |
| *SCD* | blood, brain and buccal | Hagerty et al. 2016, Witt et al. 2020 |
| *SCN1A* | blood, brain, buccal | Hagerty et al. 2016, Lohoff et al. 2018 |
| *SDCBP2* | blood, brain and buccal | Hagerty et al. 2016, Witt et al. 2020 |
| *SEC14L1* | Blood, brain and buccal | Hagerty et al. 2016, Witt al. 2020 |
| *SEC24D* | blood, brain and buccal | Hagerty et al. 2016, Witt et al. 2020 |
| *SEMA4B* | blood & bulk brain | Lohoff et al. 2018, Witt et al. 2020 |
| *SEMA4D* | blood, lymphocytes | Philibert et al. 2014, Witt et al. 2020 |
| *SLC12A8* | blood, brain and buccal | Hagerty et al. 2016, Witt et al. 2020 |
| *SLC1A2* | blood | Lohoff et al. 2020, Wilt et al. 2020 |
| *SLC26A1* | blood, brain and buccal | Hagerty et al. 2016, Witt et al. 2020 |
| *SLC2A4RG* | Blood, brain and buccal | Hagerty et al. 2016, Witt. 2020 |
| *SLC36A1* | blood & bulk brain | Lohoff et al. 2018, Witt et al. 2020 |
| *SLC39A8* | blood, brain and buccal | Hagerty et al. 2016, Witt et al. 2020 |
| *SLC6A3* | brain and buccal | Hagerty et al. 2016, Xu et al. 2017 |
| *SLIT3* | blood, brain and buccal | Hagerty et al. 2016, Witt et al. 2020 |
| *SMAD6* | Blood, brain and buccal | Hagerty et al. 2016, Witt et al. 2020 |
| *SMYD2* | blood, brain and buccal | Hagerty et al. 2016, Witt et al. 2020 |
| *SNED1* | blood & bulk brain | Lohoff et al. 2018, Witt et al. 2020 |
| *SNHG1* | blood | Lohoff et al. 2020, Wilt et al. 2020 |
| *SORL1* | blood, brain and buccal | Hagerty et al. 2016, Witt et al. 2020 |
| *SOX11* | blood, brain and buccal | Hagerty et al. 2016, Witt et al. 2020 |
| *SPTBN4* | brain and buccal | Hagerty et al. 2016, Witt et al. 2020 |
| *STAM2* | blood, post-mortem brain | Lohoff et al. 2020, Wilt et al. 2020 |
| *TBC1D16* | blood, lymphocytes | Philibert et al. 2014, Witt et al. 2020 |
| *TERT* | blood, brain and buccal | Hagerty et al. 2016, Witt et al. 2020 |
| *TMEM72-AS1* | blood | Lohoff et al. 2020, Wilt et al. 2020 |
| *TNFRSF10B* | blood, lymphocytes | Philibert et al. 2014, Witt et al. 2020 |
| *TNFRSF8* | blood, lymphocytes | Philibert et al. 2014, Witt et al. 2020 |
| *TOLLIP* | blood, post-mortem brain | Lohoff et al. 2020, Wilt et al. 2020 |
| *TP53INP2* | blood, brain and buccal | Hagerty et al. 2016, Witt et al. 2020 |
| *TPO* | blood, brain and buccal | Hagerty et al. 2016, Witt et al. 2020 |
| *TPST1* | blood, lymphocytes | Philibert et al. 2014, Witt et al. 2020 |
| *TRAPPC3* | blood | Lohoff et al. 2020, Wilt et al. 2020 |
| *TSC2* | Blood, saliva | Lohoff et al. 2020, Witt et al. 2020 |
| *TTC12* | blood | Witt et al. 2020, Xu et al. 2017 |
| *UBE2E1* | blood | Lohoff et al. 2020, Wilt et al. 2020 |
| *USP6NL* | blood | Lohoff et al. 2020, Wilt et al. 2020 |
| *UTRN* | blood | Lohoff et al. 2020, Wilt et al. 2020 |
| *WDR27* | blood, brain and buccal | Hagerty et al. 2016, Witt et al. 2020 |
| *WIF1* | blood | Lohoff et al. 2020, Wilt et al. 2020 |
| *ZDHHC19* | blood, brain and buccal | Hagerty et al. 2016, Witt et al. 2020 |
| *ZFP36L1* | blood | Lohoff et al. 2020, Wilt et al. 2020 |
| *ZMIZ2* | Blood, lymphoblasts | Philibert et al. 2014, Witt et al. 2020 |

**Supplementary Table 3. Similar Pathways Differentially Methylated Across Studies**

| **Pathway Theme** | **Relevant Pathways** |
| --- | --- |
| **Gene expression** | - Positive regulation of gene expression (Wang et al. 2016) - Negative regulation of gene expression (Liu et al. 2018) - Regulation of gene expression, epigenetic (Gatta et al. 2019, Liu et al. 2018) |
| **Calcium** | - Calcium signaling pathway (Zhang et al. 2013) - Calcium regulation in the cardiac cell (Weng et al. 2015) - Calcium ion binding (Gatta et al. 2019) - Calcium-dependent phospholipid binding (Gatta et al. 2019) |
| **Apoptosis** | - Apoptosis (Philibert et al. 2014) - Regulation of apoptosis (Philibert et al. 2014) |
| **Cell death** | - Cell death (Philibert et al. 2014) - Regulation of cell death (Philibert et al. 2014) - Negative regulation of cell death (Philibert et al. 2014) - Regulation of programmed cell death (Philibert et al. 2014) - Programmed cell death (Philibert et al. 2014) |
| **Cellular process** | - Cellular process (Liu et al. 2018, Philibert et al. 2014) - Negative regulation of cellular process (Philibert et al. 2014) - Positive regulation of cellular process (Liu et al. 2018) - Regulation of cellular process (Philibert et al. 2014, Liu et al. 2018) |
| **Biological process** | - Positive regulation of biological process (Liu et al. 2018, Witt et al. 2020) - Negative regulation of biological process (Philibert et al. 2014, Liu et al. 2018) - Regulation of biological process (Liu et al. 2018) |
| **Biosynthetic process** | - Biosynthetic process (Gatta et al. 2019) - Negative regulation of biosynthetic process (Liu et al. 2018) - Positive regulation of biosynthetic process (Wang et al. 2016) |
| **Epithelial response to bacteria** | - H. pylori epithelial signaling (Xu et al. 2019) - Bacterial invasion of epithelial cells (Xu et al. 2019) |
| **Homeostasis** | - Chemical homeostasis (Zhang et al. 2013) - Homeostatic process (Zhang et al. 2013) |
| **Immune response** | - Immune response (Witt et al. 2020, Gatta et al. 2019) - Humoral immune response, protein synthesis, cell morphology (Hagerty et al. 2016) - Cell activation involved in immune response (Witt et al. 2020) |
| **Leukocytes** | - Leukocyte differentiation (Lohoff et al. 2020) - Leukocyte activation (Witt et al. 2020) - Leukocyte transendothelial migration (Xu et al. 2019) - Leukocyte activation involved in immune response (Witt et al. 2020) - Leukocyte degranulation (Witt et al. 2020) - Myeloid leukocyte activation (Witt et al. 2020) - Myeloid leukocyte mediated immunity (Witt et al. 2020) |
| **GTPase** | - Regulation of GTPase signal transduction (Philibert et al. 2014) - Regulation of GTPase activity (Witt et al. 2020) - Regulation of small GTPase mediated signal transduction (Wang et al. 2016) - Positive regulation of GTPase activity (Witt et al. 2020) - Small GTPase mediated signal transduction (Philibert et al. 2014) |
| **Signaling** | - Regulation of signaling (Witt et al. 2020) - Regulation of signaling pathways (Philibert et al. 2014) - Signaling (Witt et al. 2020, Philibert et al. 2014) |
